# Cellular Mechanisms Responsible for Success and Failure of Bone Substitute Materials

**DOI:** 10.3390/ijms19102893

**Published:** 2018-09-23

**Authors:** Tim Rolvien, Mike Barbeck, Sabine Wenisch, Michael Amling, Matthias Krause

**Affiliations:** 1 Department of Orthopedics, University Medical Center Hamburg-Eppendorf, 20246 Hamburg, Germany; t.rolvien@uke.de; 2 Department of Oral and Maxillofacial Surgery, University Medical Center Hamburg-Eppendorf, 20246 Hamburg, Germany; mike.barbeck@uke.de; 3 Institute of Veterinary Anatomy, Histology and Embryology, Justus Liebig University of Giessen, 35385 Giessen, Germany; Sabine.Wenisch@vetmed.uni-giessen.de; 4 Department of Osteology and Biomechanics, University Medical Center Hamburg-Eppendorf, 22529 Hamburg, Germany; 5 Department of Trauma, Hand and Reconstructive Surgery, University Medical Center Hamburg-Eppendorf, 20246 Hamburg, Germany; m.krause@uke.de

**Keywords:** bone substitute, biomaterial, osteoclast, osteoblast, remodeling, bone regeneration

## Abstract

Bone grafts, i.e., autologous, allogeneic or synthetic bone substitute materials play an increasing role in reconstructive orthopedic surgery. While the indications and materials differ, it is important to understand the cellular mechanisms regarding their integration and remodeling, which are discussed in this review article. Osteoconductivity describes the new bone growth on the graft, while osteoinductivity represents the differentiation of undifferentiated cells into bone forming osteoblasts. The best case is that both mechanisms are accompanied by osteogenesis, i.e., bone modeling and remodeling of the graft material. Graft incorporation is mediated by a number of molecular pathways that signal the differentiation and activity of osteoblasts and osteoclasts (e.g., parathyroid hormone (PTH) and receptor activator of nuclear factor κβ ligand (RANKL), respectively). Direct contact of the graft and host bone as well as the presence of a mechanical load are a prerequisite for the successful function of bone grafts. Interestingly, while bone substitutes show good to excellent clinical outcomes, their histological incorporation has certain limits that are not yet completely understood. For instance, clinical studies have shown contrasting results regarding the complete or incomplete resorption and remodeling of allografts and synthetic grafts. In this context, a foreign body response can lead to complete material degradation via phagocytosis, however it may also cause a fibrotic reaction to the bone substitute. Finally, the success of bone graft incorporation is also limited by other factors, including the bone remodeling capacities of the host, the material itself (e.g., inadequate resorption, toxicity) and the surgical technique or preparation of the graft.

## 1. Introduction

For the effectiveness of orthopedic or dental implants, it is essential to create a mechanically stable interface with fusion of the implant surface and the bone tissue. Since bone defects are common problems in complex fractures, revision arthroplasty procedures or tumor reconstructions, bone substitute materials (bone grafts) are required to fill these bone defects and to ensure a tight junction between the implant and the host bone. For example, the surgical treatment of intra-articular fractures often involves bone grafts to ensure the anatomic reduction of the depressed joint fragments and to preserve the joint surface [1].

Bone grafts that are commonly used are autologous, allogeneic (cadaveric bone/bone bank) or synthetic. The main requirements for bone grafts are osteoconduction (new bone growth on the graft), osteoinduction (cells differentiating into bone forming osteoblasts) and osteogenesis (bone/callus formation). While the transplantation of autologous bone, which is commonly harvested from the iliac crest or via the Reamer-Irrigator-Aspirator, remains the gold standard providing osteoconductive and osteoinductive features, it is also associated with high donor side morbidity and limited availability [2,3]. Therefore, both allografts (including structural allografts and allograft chips/particulate bone) and synthetic grafts (including ceramics and metals) are regarded as a suitable alternative for bone regeneration. However, the regenerative potential of allografts and synthetic grafts may be limited to osteoconductive bone growth, which is why bone substitute materials with additional osteoinductive features have been developed.

The combination of bone grafts with cells (e.g., osteoblasts, mesenchymal stem cells, platelet-rich plasma) or proteins (e.g., collagen, bone morphogenetic protein) enables the promotion of cell adhesion on osteoconductive material to create osteoinductive materials [4,5]. Another strategy to overcome the issue of limited osteoinductivity is the development of novel tissue, perfectly engineered “biomimetic” materials. Also, the combination of bone grafts with bioactive metal ions has been proposed for improved bone regeneration [6].

**Figure 1:**
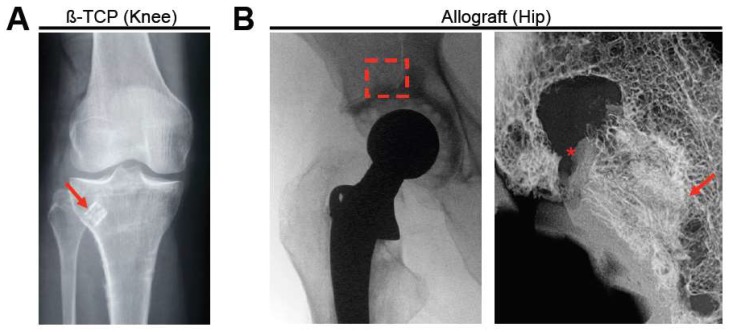
Clinical examples (radiographs) of two different bone substitute types. (**A**) beta-tricalcium phosphate (β-TCP) block (red arrow) used in a patient with a multi-fragment proximal tibia fracture. Adapted from [1]. (**B**) Implantation of a structural allograft in revision total hip arthroplasty, left: clinical radiograph (red box indicates the area where the allograft was implanted), right: post-mortem high-resolution contact radiograph. * indicates absent area with no contact, arrow indicates close contact between the graft and the host bone.

During the clinical use of bone substitutes, the success of their incorporation can be readily estimated by conventional radiography (Figure 1). However, research studies require a closer examination, e.g., by microscopic preparations. Several histological and micro-morphological studies from retrieved specimens have proven the successful incorporation of bone substitutes such as allografts or synthetic grafts [1,7,8]. For allografts, bone remodeling with the subsequent interconnection of the host bone and the graft bone was found in the majority of the interface, leading to the progressive incorporation of the bone graft [9]. Furthermore, synthetic materials such as beta-tricalcium phosphate (β-TCP) were also found to induce bone formation and promote bone repair [1]. While the restoration of bone defects is often successful, there are also certain materials and conditions which are associated with a failure of the bone healing process, for example in terms of impaired integration and induction of immunologic responses [10]. The following article reviews the current state of knowledge about the success and failure of bone substitute materials, and will be focused on the cellular basis and histological features of the bone substitute incorporation process.

## 2. Bone Remodeling

The skeleton is normally subject to constant remodeling which is mediated by the activity of two different cell types, the bone-forming osteoblasts and the bone-resorbing osteoclasts [11]. Osteoblastogenesis is initiated by the differentiation of the mesenchymal stem cells into osteoprogenitors and is associated with the high expression of hormones and cytokines, such as parathyroid hormone (PTH), interleukins, insulin-like growth factor 1 (IGF-1) and transforming growth factor beta (TGF-β). After proliferation, the bone-forming cells express alkaline phosphatase (ALP) and secrete collagen type 1 and other matrix proteins before the matrix becomes mineralized. Osteoclasts are large multinucleated terminally differentiated cells from the monocyte-macrophage lineage that are able to resorb bone. While excessive bone resorption leads to bone loss (i.e., osteoporosis, tumor osteolysis, etc.), it is also needed for the renewal of the skeleton and for graft incorporation (as discussed later in this article). Many factors signal osteoclast development where some of the most important early signals are RANKL (receptor activator of nuclear factor κβ ligand) and M-CSF (Macrophage colony-stimulating factor).

This skeletal remodeling process is influenced by osteocytes, which represent terminal differentiated osteoblasts, and form a cellular network within the mineralized bone matrix [12]. While the bone remodeling process involves bone formation following bone resorption, the bone modeling process only involves the formation of new bone. Bone modeling is a prevailing process during growth, modifying the shape and size of the bone. Bone remodeling is a lifelong process that persists throughout life, maintaining bone function by continuously replacing old bone with new bone.

The concept of bone remodeling is not only important regarding common and uncommon skeletal diseases such as osteoporosis, however it is also important in the understanding of bone healing as well as the success and failure of bone substitutes. Bone (fracture) healing enables the full restoration of fractured or damaged bone to its previous composition, structure and function [13]. One may differentiate primary and secondary bone healing. This means a direct continuous ossification of small fracture gaps or indirect ossification through multiple events such as blood clotting, inflammation, cartilage callus formation, intramembranous and endochondral ossification and eventually bone remodeling. Large gaps and/or other conditions (e.g., infections, impaired blood supply) may lead to the insufficient healing of bone defects, ultimately resulting in non-union. In general, a length exceeding 2–2.5 times the diameter of the affected bone was found to be problematic for spontaneous fracture healing (“critical sized defect”) [14]. Bone substitutes come into play to provide bridging and to improve the bone regeneration of bone defects that have arisen from large fracture gaps or from complex orthopedic reconstructions.

## 3. Basic Cellular Concept of Bone Substitute Incorporation

Bone substitute materials vary widely, while all of them have individual advantages and disadvantages. Apart from autografts and allografts, synthetic bone grafts include hydroxyapatite [15], glass ceramics [16], polylactic acid/polyglycolic acid polymers [17], demineralized bone [18] and the most commonly used calcium phosphates [19]. It is important to note that the basic cellular mechanisms of their incorporation are always similar and involve osteoclastic bone resorption followed by new osteoblastic bone formation (i.e., remodeling). While osteoblasts are considered as the main players in osseointegration, increasing evidence suggests that osteoclasts are of crucial importance for the durability of the different biomaterials. In other words, a preferred characteristic of these materials is their ability to be remodeled through the activity of both bone cell types [20]. Therefore, the extent of the graft incorporation is always mediated by the resorbability of the graft as well as the achievement of sufficient bone formation on the graft. The success and activity of osteoclastic bone resorption can be visualized by specific immunohistochemical staining methods, e.g., tartrate-resistant acid phosphatase (TRAP) staining [21]. Besides the requirement of the graft to be remodeled, the perigraft environment and the mechanical environment are some of the key factors affecting the graft incorporation [22].

**Figure 2:**
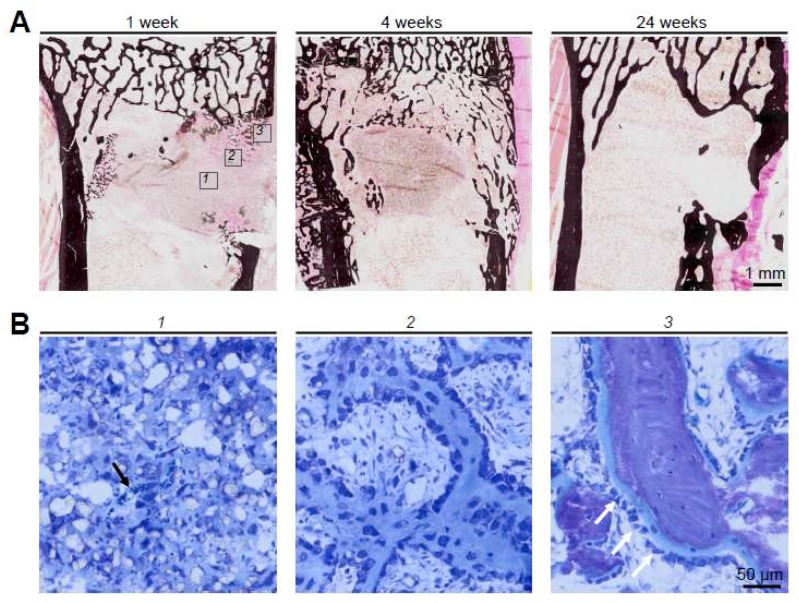
Remodeling of β-TCP (Cerasorb^®^) in rabbits [23]. (**A**) Von Kossa stained sections demonstrating the healing of the surgically induced tibial defect from the formation of intramedullary bone after 4 weeks to a new cortex until 24 weeks. Boxs 1–3 indicate the regions of interest that are shown at higher magnification below. (**B**) At higher magnification and in toluidine blue sections, the cellular process of the remodeling process can be reconstructed. (1): β-TCP granules and invasion by blood vessels (black arrow); (2) formation of immature, unmineralized bone; and (3) bone trabeculae and bone formation by osteoblasts (white arrows).

The basic cellular mechanism of bone substitute remodeling can be described in multiple steps and is a well-studied process. First, a hematoma forms around the implanted graft. Secondly, necrosis of the graft occurs followed by an inflammatory response and the formation of a fibrovascular stroma. Thirdly, blood vessels and osteogenic precursor cells infiltrate the graft. Finally, new bone formation (and potentially bone resorption) is initiated, indicating successful (re)modeling of the graft. On a molecular level, the activation of osteoblasts is mediated through osteoblast related transcription factors (e.g., Runx2). Osteoclast activation is directly linked to osteoblast function as well as the immune response that is mediated by cytokines (e.g., RANKL). An overview of this process from a histologic point of view which is based on a previous study of β-TCP combined with hyaluronic acid and methylcellulose [23] can be found in Figure 2.

The fact that the graft incorporation can be promoted by growth factors such as bone morphogenetic protein (BMP), stromal stem cells or platelet-rich plasma has been shown multiple times [24] and shows how these factors extend the function of osteoconductivity of the bone substitute towards osteoinductivity through inducing the bone modeling and remodeling cascade. In particular, increased bone formation and better vascular invasion have been found as a consequence of growth factor administration [24]. Also, glycosaminoglycans, such as hyaluronic acid, supported the osteoconductive effect of synthetic bone graft materials [23], and this effect was shown to be mediated by an upregulation of BMP-2 activity [25]. Importantly, daily administration of PTH (teriparatide, PTH 1-34) also improved osseointegration through the stimulation of bone formation [26], signifying its potential use as a drug for the treatment of delayed union and graft incorporation.

Regarding the perigraft environment, it is clear that sufficient vascularization (and vascular invasion) and a germ-free environment are the absolute minimum requirements for bone regeneration to take place. However, as bone substitutes are commonly used in old patients with compromised bone status (i.e., osteoporosis), the overall dissociation of low bone formation and increased bone resorption might also limit the local potential of bone incorporation. The (long-term) integrity of the graft is primarily influenced by the mechanical stress. According to the well-known and often confirmed theory by Julius Wolff (Wolff’s law) [27], bone adapts to mechanical stress. There is repeated evidence that osteocytes are the cells that act as mechanosensors to trigger bone remodeling at the bone surface [28]. Therefore, the absence of mechanical load leads to bone resorption rather than an adequate remodeling of the graft [29].

## 4. Allograft Incorporation

Frequently used allografts mainly include structural allografts or allograft chips. Histological studies on cancellous allograft chips in humans have shown that they are completely incorporated, forming a new bone structure [8]. More specifically, a revascularization of the graft was followed by osteoclastic resorption and new bone formation on the graft. In structural allografts, successful incorporation and remodeling was also detected [9], however, this was most likely limited to the area of direct contact between the host bone and the allograft bone [30]. In general, the difference in the remodeling of chips and structural allografts may be explained by the different surgical techniques (i.e., impaction grafting vs. maintained structure). Due to the larger surface areas of allograft chips in comparison to structural allografts, they have advantages regarding access to the bone cells (i.e., osteoblasts and osteoclasts) and may therefore have improved the osteoconductive capacities [31]. Although structural allografts are most likely a better alternative for larger bone defects, it is still not clear (both clinically and in basic research) which defect sizes should not be exceeded for allograft chips in order to guarantee a sufficient incorporation. Regarding the time course of allograft incorporation, it was found that allograft chips had incorporated within the first 12 weeks after implantation [32]. In structural allografts, long-term follow up observations revealed no time-dependent increase in the incorporation between four years and 22 years after implantation [9].

**Figure 3:**
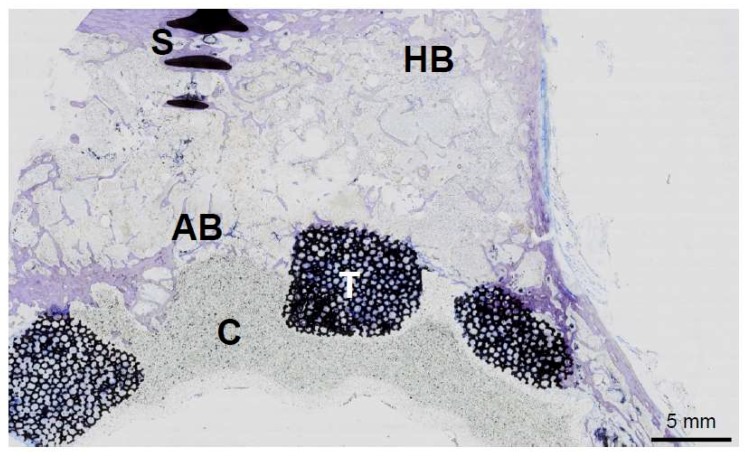
The ground section of an acetabulum that was explanted from a patient years after revision arthroplasty. HB: host bone AB: allograft bone, S: screw, T: tantalum augmentation, C: bone cement. The allograft has been incorporated by the host bone, while additional bone growth on the tantalum can be seen.

**Figure 4:**
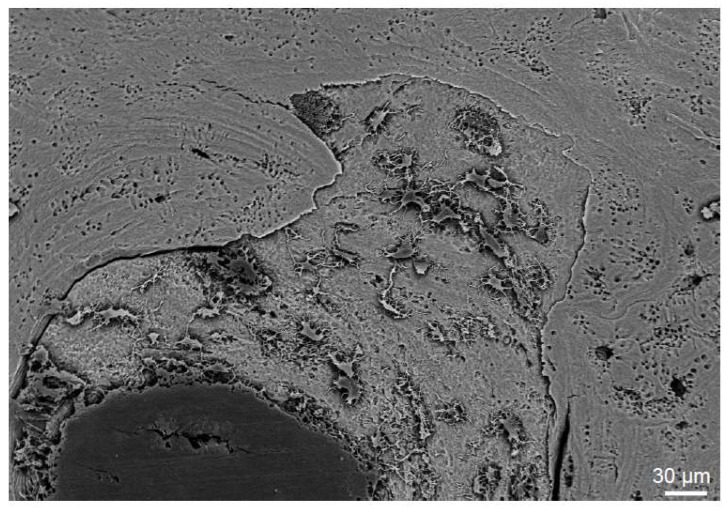
Scanning electron microscopy (SEM) image of an acid-etched human allograft bone specimen, demonstrating the interface of the dead allograft bone and the new bone growth with viable and connected osteocytes on the graft.

The extent of allograft incorporation within the host bone can be determined in ground sections of total hip explants (Figure 3), for example. The main advantage of this technique is that screws and implants, such as tantalum augmentations, can also be visualized in order to estimate the full area of incorporation (Figure 3). These mechanical barriers result in an absent local remodeling with insufficient incorporation of the graft around them [9]. On a higher magnified scale, the osteoconductive ability of allografts can be demonstrated by various other techniques. In scanning electron microscopy of acid-etched plastic embedded bone specimens from previously implanted allografts, the new bone formation on the allograft surface can be visualized (Figure 4). Here, osteocyte lacunae that are connected via canaliculi are the hallmark of viable bone matrix that stands in contrast to low or no connection of osteocyte lacunae within the allograft.

## 5. Requirements for Synthetic Bone Substitutes

Modern requirements for synthetic bone substitute materials include biocompatibility, biodegradation and osteoconductivity rather than just a filling of bone defects. Importantly, the bone substitute should provide structural support to the newly formed bone tissue. In other words, it should serve as a template for bone cell attachments and the subsequent formation of the extracellular matrix [33,34]. Further requirements include the possibility to provide direct contact between the host bone and the graft, as well as the colonization of the graft by host blood vessels.

It is known that bone formation and bone resorption are influenced by individual properties of the biomaterial, such as structural morphology, porosity and particle size [21,35]. While bone degradation occurs faster with small granules (<50 µm), bone regeneration may be improved through slower degradation in larger granules (>500 µm), as shown for TCP [21]. In addition to the size, the purity of the compounds influences bone regeneration. Impurities can potentially weaken the scaffolds through the increased risk of particular disintegration. Given the need for a close contact of the bone substitute and the host bone, despite the presence of irregularly shaped bone defects, various modifications such as granules or block shapes are available. Furthermore, injectable bone substitutes have been developed to avoid unnecessary preparation of the graft and host bone leading to additional bone loss [36].

Since bone substitutes have been commonly used in delayed fracture healing and/or infected areas, they may also serve as carriers for growth factors or antibiotics. Next to osteoconductive bone substitutes, growth factors are one of the minimum requirements that have to be present for successful bone repair [37]. Therefore, these bone grafts should facilitate cell attachment and migration and should incorporate desirable biological and chemical signaling. Furthermore, the successful use of ceramics that are composed of different hydroxyapatite to tricalcium phosphate ratios as carriers for growth factors (e.g., BMP) has been studied and confirmed [38]. However, the development of a perfect osteoconductive, osteoinductive and osteogenic tissue-engineered product is still being studied [34].

In infectious conditions, the interest is focused on bone substitute materials which can release antibiotics. Several methods have been described for loading porous ceramics with additives like antibiotics or other drugs [39,40]. Thereby, biodegradable bone substitutes may be preferred as they do not have to be removed surgically.

Ceramic-based synthetic bone substitutes are completely resorbable by osteoclasts, and although weaker than cortical bone, they have proved to be effective by replacement through new bone [19]. However, one main limitation of these materials is their brittle nature and poor mechanical properties.

## 6. Bone Modeling on Implants

Due to the poor mechanical features of most synthetic bone substitutes (as stated above), they are mostly limited to non-load-bearing applications. Given their better mechanical strength compared to bone substitutes, metals (e.g., titanium) have shown to have a greater potential as the basis of implants for long-term load-bearing orthopedic applications. Bone ingrowth around various types of implants represents a desirable feature for long-term optimal stability. Various orthopedic implants have been tested for their integration within the host bone. Porous coated implants have been considered suitable for the ingrowth of bone [41]. It is interesting to note that similarly to bone substitute materials, the ingrowth into porous implants is again influenced by factors such as the porous structure, implant stiffness or micromotion between the implant and the host bone [42,43]. This illustrates that bone cells need a certain environment, including sufficient surface areas and mechanical stability, regardless of whether bone grafts or metal implants have been used.

**Figure 5:**
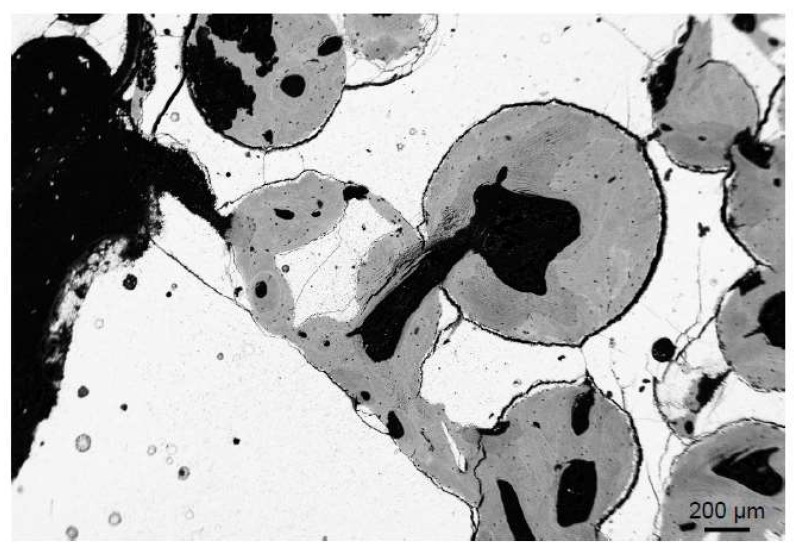
Bone ingrowth into a porous-coated tantalum implant (human). Image acquired by backscattered electron microscopy.

Metal implants are most commonly used in different joint replacement surgeries, and porous coating is especially important considering the growing numbers of cementless procedures. However, different implants and prosthetic augmentations may also be used in the treatment of extensive bone loss that cannot always be treated with bone grafts [44]. Tantalum augmentations have been shown to provide a good substrate for bone attachment in several in vitro and animal studies [45,46], and in vivo studies have shown promising clinical and radiographic results [47] as well as the formation and ingrowth of bone, even under difficult conditions [48]. In Figure 5, the successful bone growth on the porous tantalum is demonstrated (Figure 5).

## 7. Limits and Failure of Bone Substitutes and Implants

**Figure 6:**
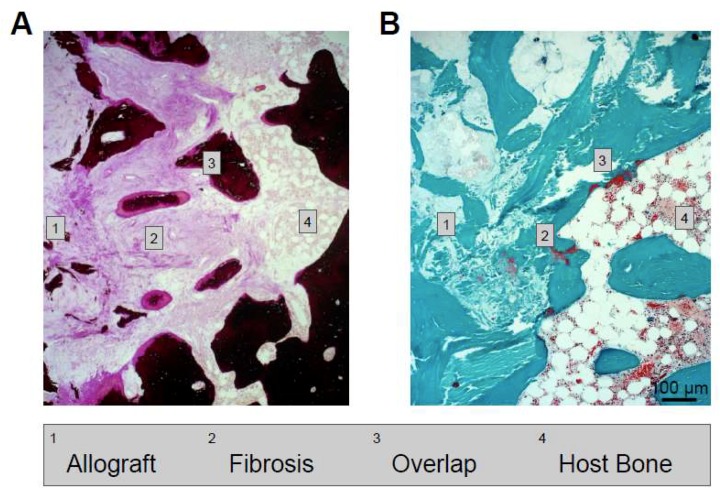
Histological sections demonstrating the interface of the allograft and host bone that is composed of different zones, including fibrosis and overlap (1–4). (**A**) Von Kossa staining. (**B**) Trichrome Goldner staining.

The incorporation of bone substitutes also has certain limits. For example, when observing the interface between the host bone and the allograft bone, not only can an overlap be seen, but also a layer of fibrosis containing blood vessels (Figure 6). Furthermore, while superficial areas of structural allografts are often completely remodeled, the center of the allograft remains unremodeled. Therefore, despite the very good clinical outcome of these allografts, as well as the impression of a completely remodeled graft in conventional imaging [49,50,51,52], there are indications that the incorporation might be less pronounced than previously expected (at least in humans) [9]. Also, synthetic materials showed incomplete remodeling with detectable remnants in human studies [1], although various animal studies indicated a complete remodeling [53].

**Figure 7:**
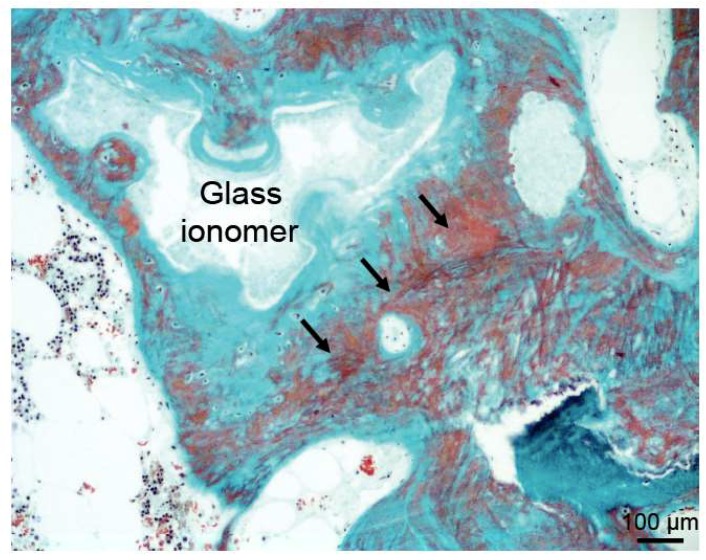
Image of a trichrome Goldner stained section showing bone growth around glass ionomer cement. However, this bone is characterized by large areas of non-mineralized bone (osteoid, black arrows), contributing to insufficient bone stability.

Apart from the described conditions that involve incomplete remodeling but sufficient osseointegration, there are also cases of true failure of bone substitutes. These failures include mechanical failure [54], absence of integration or graft collapse and an inadequate immune response (29). The latter is described in detail in the next paragraph. There are also other reasons for the failure of bone substitutes. Glass ionomer cement was used as a bone substitute in granulate form, mixed with homologous bone. While initially excellent biocompatibility was reported, there were several cases with a failure of the bone substitute in terms of early loosening of the prosthesis. Histology indicated that osteoblastic function and bone mineralization were clearly inhibited [55]. In fact, large areas of non-mineralized osteoid matrix were seen in undecalcified histological sections, pointing to local osteomalacia (Figure 7). These areas of non-mineralized bone had not been associated with glass ionomer due to the decalcification of the tissue specimens. Further examination showed large deposits of aluminum in the adjacent connective tissue and bone as the cause for the absent mineralization of the newly formed bone tissue. These cases also show that the appropriate methodology (i.e., undecalcified bone preparation) is of paramount importance to unravel mineralization defects and should therefore be the method of choice in analyzing the incorporation of bone grafts.

## 8. Immune Responses to Bone Substitute Materials

**Figure 8:**
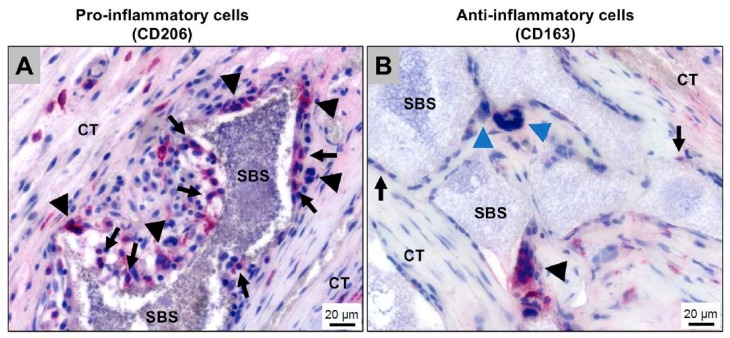
Histological images showing the immunologic alignment of both macrophages and multinucleated giant cells (MNGCs) within the bony implantation bed of a synthetic bone substitute (SBS). CT: connective tissue. (**A**) Detection of pro-inflammatory mononucleated (arrows) and multinucleated cells (arrowheads) at the bone substitute granule surfaces (CD206-immunostaining); (**B**) Detection of anti-inflammatory mononuclear (arrows) and multinucleated (black arrowhead) cells at the bone substitute granule surfaces. Interestingly, most of the MNGCs that were adherent to the bone substitute granules (blue arrowheads) did not show an expression of the anti-inflammatory molecule (CD163-immunostaining).

**Figure 9:**
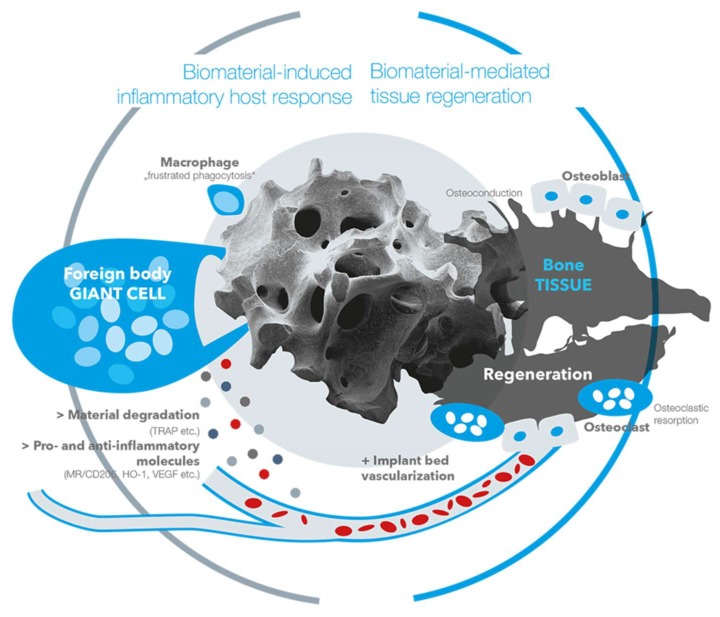
Theoretical pathways between the biomaterial-induced inflammatory host response and the material-mediated tissue regeneration.

The inflammatory tissue reactions to biomaterials in general, and also to bone substitute materials, have shown to have an eminent influence on healing processes [56,57]. In this context, it has been revealed that every biomaterial elicits a material-specific tissue reaction cascade that is called a “foreign body reaction to biomaterials” [57]. This cascade starts with an agglomeration of proteins on the surfaces of a biomaterial within the first seconds to minutes after implantation [58]. Interestingly, the protein layer is dependent on the physicochemical properties of a biomaterial, such as the surface chemistry or the surface topography [57,58]. Thus, all of the material properties lead to the agglomeration of a material-specific protein layer which is not only specifically related to the bound proteins, however it is also related to the conformation of the proteins [57,58]. Altogether, the proteins and different binding sites, such as the RGD motif in case of the fibrinogen molecule, mediate between the biomaterial and the first generation of cells within an implantation site [57,58,59]. Cell types such as monocytes, macrophages and neutrophils interact with the proteins and a material-specific release of cytokines that guide the further tissue reaction pattern that is released [57,58]. In the further course of the tissue reaction cascade, regulatory cell types such as macrophages and multinucleated giant cells (MNGCs), which are polykaryons of monocytes or macrophages, are involved [57,60,61,62,63,64]. Even in cases of bone substitutes and the related cell reactions, these cells are of special interest for the material-related healing success or implant failures [60,65]. Both cell types have been identified as key regulators of the degradation process of bone substitute materials and the pro- and anti-inflammatory tissue response [60,65,66]. When macrophages have a restricted phagocytosis capacity, they fuse into MNGCs [67]. Interestingly, not only have MNGCs been shown to be of the foreign body giant cell phenotype instead of being osteoclastic polykaryons, however they have also been shown to express pro- and anti-inflammatory molecules within the implant bed of both synthetic and xenogeneic bone substitutes (Figure 8) [60,63]. These results have led to the assumption that MNGCs are a heterogeneous cell population that is comparable to anti-inflammatory M1- and pro-inflammatory M2-macrophages (Figure 8) [60]. In this context, it has been shown by Ghanaati et al. that this multinucleated cell type is able to support implant bed vascularization as well as bone cell differentiation and bone growth by the expression of the vascular endothelial growth factor (VEGF) [68]. Interestingly, different material properties have shown to influence the number of MNGCs and also affect the expression pattern of pro- and anti-inflammatory molecules [61,62,64,68,69]. Although the precise relationship between the different physicochemical properties of a bone substitute and the activation or expression pattern of MNGCs is unknown, these data lead to the conclusion that a special combination of material properties can optimize the immunologic response to a bone substitute to optimally support the bone healing process (Figure 9). Altogether, the immunologic response is strongly connected to the process of bone healing (Figure 9).

Recent results from an in vivo study of Barbeck et al. [70] reveal that material degradation is mainly carried out by pro-inflammatory cells of the macrophage and MNGC lines (Figure 8). These results lead to the assumption that the degradation of bone substitutes is strongly connected to pro-inflammatory cell reactions. This assumption is further reinforced by the fact that the degradation of bone substitutes is often accompanied by the synthesis of reactive oxygen species (ROS) that have shown to play an important role in the progression of inflammatory conditions [71]. In addition, it is questionable whether the local pro-inflammatory milieu that was induced by the material degradation was compensated by an equally high anti-inflammatory tissue response, or if a balanced result of pro- and anti-inflammation was important for the course of bone healing (Figure 8). However, the material-induced immune response could also lead to a regenerative failure as the foreign body response to a bone substitute can end in its fibrous encapsulation [72,73]. In other words, the foreign body response that can lead to complete material degradation via phagocytosis might also cause a fibrotic reaction to a bone substitute. In this context, it is conceivable that after the initial frustrated phagocytosis of macrophages and the following induction of MNGCs, these polykaryons are incapable of degrading the bone substitute. This process might lead to the expression of molecules such as the platelet-derived growth factor (PDGF) and transforming growth factor beta (TGF-β), matrix metalloproteinases MMP-2 and -9 or platelet-derived growth factor BB (PDGF-BB) that lead to capsule formation by myofibroblasts [74,75]. These molecules are suspected to lead not only to the differentiation of fibroblasts to myofibroblasts, but also to the differentiation of other cell types such as epithelial cells, smooth muscle cells, fibrocytes or macrophages [76,77,78,79]. This reactivity is also dependent on the bone substitute material that is used [73]. In the case of fibrous encapsulation of a bone substitute, the material-related inflammatory tissue reaction is downsized as the material is isolated, and further interaction between the host and implanted device is limited [80]. Interestingly, a thin fibrous capsule seems to be tolerable in the process of bone regeneration, while an exaggerated inflammatory process that ends in the manifestation of a thick fibrous capsule can be considered a tissue reaction that is associated with restricted bioincompatibility [81].

Moreover, a material-related immunological response that includes an exaggerated level of pro-inflammation, and particularly one that combines such a high level of inflammation with a high level of dissolution of the bone substitute, will often lead to implant failures as the material cannot fulfill its role as an osteoconductive scaffold in the framework of the bone regeneration process [82].

## 9. Conclusions

Understanding the basic cellular mechanisms of bone healing and bone substitute integration is not only important for further advancement with these materials, however it is also important for orthopedic surgeons who must choose both the most suitable graft and technique for implantation. The successful incorporation of bone grafts and implants involves remodeling by osteoblasts and osteoclasts and is influenced by a number of factors including the perigraft environment (e.g., vascularity and sterility), mechanical stability and growth factors. The way that the body’s immune response can lead to bone substitute material degradation and subsequent successful incorporation on one hand and a fibrotic reaction on the other hand merits further investigation.

## References

[1] Rolvien T., Barvencik F., Klatte T.O., Busse B., Hahn M., Rueger J.M., Rupprecht M. (2017). β-TCP bone substitutes in tibial plateau depression fractures. Knee.

[2] Sen M.K., Miclau T. (2007). Autologous iliac crest bone graft: Should it still be the gold standard for treating nonunions?. Injury.

[3] Dawson J., Kiner D., Gardner W., Swafford R., Nowotarski P.J. (2014). The reamer-irrigator-aspirator as a device for harvesting bone graft compared with iliac crest bone graft: Union rates and complications. J. Orthop. Trauma.

[4] Anselme K. (2000). Osteoblast adhesion on biomaterials. Biomaterials.

[5] Kruyt M.C., van Gaalen S.M., Oner F.C., Verbout A.J., de Bruijn J.D., Dhert W.J. (2004). Bone tissue engineering and spinal fusion: The potential of hybrid constructs by combining osteoprogenitor cells and scaffolds. Biomaterials.

[6] Glenske K., Donkiewicz P., Kowitsch A., Milosevic-Oljaca N., Rider P., Rofall S., Franke J., Jung O., Smeets R., Schnettler R. (2018). *.* Applications of metals for bone regeneration. Int. J. Mol. Sci..

[7] Buma P., Lamerigts N., Schreurs B.W., Gardeniers J., Versleyen D., Slooff T.J. (1996). Impacted graft incorporation after cemented acetabular revision. Histological evaluation in 8 patients. Acta Orthop. Scand..

[8] Van der Donk S., Buma P., Slooff T.J., Gardeniers J.W., Schreurs B.W. (2002). Incorporation of morselized bone grafts: A study of 24 acetabular biopsy specimens. Clin. Orthop. Relat. Res..

[9] Butscheidt S., Moritz M., Gehrke T., Puschel K., Amling M., Hahn M., Rolvien T. (2018). Incorporation and remodeling of structural allografts in acetabular reconstruction: Multiscale, micro-morphological analysis of 13 pelvic explants. J. Bone Jt. Surg. Am..

[10] Remes A., Williams D.F. (1992). Immune response in biocompatibility. Biomaterials.

[11] Zaidi M. (2007). Skeletal remodeling in health and disease. Nat. Med..

[12] Bellido T. (2014). Osteocyte-driven bone remodeling. Calcif. Tissue Int..

[13] Einhorn T.A., Gerstenfeld L.C. (2015). Fracture healing: Mechanisms and interventions. Nat. Rev. Rheumatol..

[14] Gugala Z., Lindsey R.W., Gogolewski S. (2007). New approaches in the treatment of critical-size segmental defects in long bones. Macromol. Symp..

[15] Uchida A., Nade S., McCartney E., Ching W. (1985). Bone ingrowth into three different porous ceramics implanted into the tibia of rats and rabbits. J. Orthop. Res..

[16] Berger G., Gildenhaar R., Ploska U. (1995). Rapid resorbable, glassy crystalline materials on the basis of calcium alkali orthophosphates. Biomaterials.

[17] Athanasiou K.A., Niederauer G.G., Agrawal C.M. (1996). Sterilization, toxicity, biocompatibility and clinical applications of polylactic acid/polyglycolic acid copolymers. Biomaterials.

[18] Kaban L.B., Glowacki J. (1984). Augmentation of rat mandibular ridge with demineralized bone implants. J. Dent. Res..

[19] Campana V., Milano G., Pagano E., Barba M., Cicione C., Salonna G., Lattanzi W., Logroscino G. (2014). Bone substitutes in orthopaedic surgery: From basic science to clinical practice. J. Mater. Sci. Mater. Med..

[20] Schilling A.F., Linhart W., Filke S., Gebauer M., Schinke T., Rueger J.M., Amling M. (2004). Resorbability of bone substitute biomaterials by human osteoclasts. Biomaterials.

[21] Gauthier O., Bouler J.M., Weiss P., Bosco J., Aguado E., Daculsi G. (1999). Short-term effects of mineral particle sizes on cellular degradation activity after implantation of injectable calcium phosphate biomaterials and the consequences for bone substitution. Bone.

[22] Stevenson S., Emery S.E., Goldberg V.M. (1996). Factors affecting bone graft incorporation. Clin. Orthop. Relat. Res..

[23] Krause M., Oheim R., Catala-Lehnen P., Pestka J.M., Hoffmann C., Huebner W., Peters F., Barvencik F., Amling M. (2014). Metaphyseal bone formation induced by a new injectable beta-tcp-based bone substitute: A controlled study in rabbits. J. Biomater. Appl..

[24] Lucarelli E., Fini M., Beccheroni A., Giavaresi G., Di Bella C., Aldini N.N., Guzzardella G., Martini L., Cenacchi A., Di Maggio N. (2005). Stromal stem cells and platelet-rich plasma improve bone allograft integration. Clin. Orthop. Relat. Res..

[25] Kawano M., Ariyoshi W., Iwanaga K., Okinaga T., Habu M., Yoshioka I., Tominaga K., Nishihara T. (2011). Mechanism involved in enhancement of osteoblast differentiation by hyaluronic acid. Biochem. Biophys. Res. Commun..

[26] Daugaard H., Elmengaard B., Andreassen T.T., Baas J., Bechtold J.E., Soballe K. (2011). The combined effect of parathyroid hormone and bone graft on implant fixation. J. Bone Jt. Surg. Br..

[27] Wolff J. (1892). Das gesetz der transformation der knochen.

[28] Bonewald L.F. (2006). Mechanosensation and transduction in osteocytes. Bonekey Osteovision.

[29] Bauer T.W., Muschler G.F. (2000). Bone graft materials. An overview of the basic science. Clin. Orthop. Relat. Res..

[30] Hooten J.P., Engh C.A., Heekin R.D., Vinh T.N. (1996). Structural bulk allografts in acetabular reconstruction. Analysis of two grafts retrieved at post-mortem. J. Bone Jt. Surg. Br..

[31] Malinin T.I., Carpenter E.M., Temple H.T. (2007). Particulate bone allograft incorporation in regeneration of osseous defects; importance of particle sizes. Open Orthop. J..

[32] Schimmel J.W., Buma P., Versleyen D., Huiskes R., Slooff T.J. (1998). Acetabular reconstruction with impacted morselized cancellous allografts in cemented hip arthroplasty: A histological and biomechanical study on the goat. J. Arthroplasty.

[33] Lanza R., Langer R., Vacanti J. (1993). Tissue engineering. Science.

[34] Khan W.S., Rayan F., Dhinsa B.S., Marsh D. (2012). An osteoconductive, osteoinductive, and osteogenic tissue-engineered product for trauma and orthopaedic surgery: How far are we?. Stem Cells Int..

[35] Tadic D., Epple M. (2004). A thorough physicochemical characterisation of 14 calcium phosphate-based bone substitution materials in comparison to natural bone. Biomaterials.

[36] Low K.L., Tan S.H., Zein S.H.S., Roether J.A., Mouriño V., Boccaccini A.R. (2010). Calcium phosphate-based composites as injectable bone substitute materials. J. Biomed. Mater. Res. Part B Appl. Biomater..

[37] Giannoudis P.V., Einhorn T.A., Marsh D. (2007). Fracture healing: The diamond concept. Injury.

[38] Alam M.I., Asahina I., Ohmamiuda K., Takahashi K., Yokota S., Enomoto S. (2001). Evaluation of ceramics composed of different hydroxyapatite to tricalcium phosphate ratios as carriers for rhbmp-2. Biomaterials.

[39] Seidenstuecker M., Ruehe J., Suedkamp N.P., Serr A., Wittmer A., Bohner M., Bernstein A., Mayr H.O. (2017). Composite material consisting of microporous β-tcp ceramic and alginate for delayed release of antibiotics. Acta Biomater..

[40] Faigle G., Bernstein A., Suedkamp N., Mayr H., Peters F., Huebner W., Seidenstuecker M. (2018). Release behavior of van from four types of cap-ceramic granules using various loading methods at two different degrees of acidity. J. Mater. Sci. Mater. Med..

[41] Bobyn J.D., Pilliar R.M., Binnington A.G., Szivek J.A. (1987). The effect of proximally and fully porous-coated canine hip stem design on bone modeling. J. Orthop. Res..

[42] Li J.P., Habibovic P., van den Doel M., Wilson C.E., de Wijn J.R., van Blitterswijk C.A., de Groot K. (2007). Bone ingrowth in porous titanium implants produced by 3d fiber deposition. Biomaterials.

[43] Konttinen Y.T., Zhao D., Beklen A., Ma G., Takagi M., Kivela-Rajamaki M., Ashammakhi N., Santavirta S. (2005). The microenvironment around total hip replacement prostheses. Clin. Orthop. Relat. Res..

[44] Mabry T.M., Hanssen A.D. (2007). The role of stems and augments for bone loss in revision knee arthroplasty. J. Arthroplasty.

[45] Levine B.R., Sporer S., Poggie R.A., Della Valle C.J., Jacobs J.J. (2006). Experimental and clinical performance of porous tantalum in orthopedic surgery. Biomaterials.

[46] Bobyn J.D., Toh K.K., Hacking S.A., Tanzer M., Krygier J.J. (1999). Tissue response to porous tantalum acetabular cups: A canine model. J. Arthroplasty.

[47] Unger A.S., Duggan J.P. (2011). Midterm results of a porous tantalum monoblock tibia component clinical and radiographic results of 108 knees. J. Arthroplasty.

[48] Breer S., Hahn M., Kendoff D., Krause M., Koehne T., Haasper C., Gehrke T., Amling M., Gebauer M. (2012). Histological ex vivo analysis of retrieved human tantalum augmentations. Int. Orthop..

[49] Knight J.L., Fujii K., Atwater R., Grothaus L. (1993). Bone-grafting for acetabular deficiency during primary and revision total hip arthroplasty. A radiographic and clinical analysis. J. Arthroplasty.

[50] Deakin D.E., Bannister G.C. (2007). Graft incorporation after acetabular and femoral impaction grafting with washed irradiated allograft and autologous marrow. J. Arthroplasty.

[51] Ullmark G., Sorensen J., Nilsson O. (2009). Bone healing of severe acetabular defects after revision arthroplasty. Acta Orthop..

[52] Schreurs B.W., Keurentjes J.C., Gardeniers J.W., Verdonschot N., Slooff T.J., Veth R.P. (2009). Acetabular revision with impacted morsellised cancellous bone grafting and a cemented acetabular component: A 20- to 25-year follow-up. J. Bone Jt. Surg. Br..

[53] Johnson K.D., Frierson K.E., Keller T.S., Cook C., Scheinberg R., Zerwekh J., Meyers L., Sciadini M.F. (1996). Porous ceramics as bone graft substitutes in long bone defects: A biomechanical, histological, and radiographic analysis. J. Orthop. Res..

[54] Linhart W., Briem D., Amling M., Rueger J., Windolf J. (2004). Mechanical failure of porous hydroxyapatite ceramics 7.5 years after implantation in the proximal tibial. Der Unfallchirurg.

[55] Engelbrecht E., von Foerster G., Delling G. (2000). Ionogran in revision arthroplasty. J. Bone Jt. Surg. Br..

[56] Mountziaris P.M., Mikos A.G. (2008). Modulation of the inflammatory response for enhanced bone tissue regeneration. Tissue Eng. Part B Rev..

[57] Anderson J.M., Rodriguez A., Chang D.T. (2008). Foreign body reaction to biomaterials. Seminars in Immunology.

[58] Hu W.J., Eaton J.W., Ugarova T.P., Tang L. (2001). Molecular basis of biomaterial-mediated foreign body reactions. Blood.

[59] Tang L., Eaton J.W. (1993). Fibrin(ogen) mediates acute inflammatory responses to biomaterials. J. Exp. Med..

[60] Barbeck M., Motta A., Migliaresi C., Sader R., Kirkpatrick C.J., Ghanaati S. (2016). Heterogeneity of biomaterial-induced multinucleated giant cells: Possible importance for the regeneration process?. J. Biomed. Mater. Res. A.

[61] Barbeck M., Udeabor S., Lorenz J., Schlee M., Holthaus M.G., Raetscho N., Choukroun J., Sader R., Kirkpatrick C.J., Ghanaati S. (2015). High-temperature sintering of xenogeneic bone substitutes leads to increased multinucleated giant cell formation: In vivo and preliminary clinical results. J. Oral Implantol..

[62] Barbeck M., Udeabor S.E., Lorenz J., Kubesch A., Choukroun J., Sader R.A., Kirkpatrick C.J., Ghanaati S. (2014). Induction of multinucleated giant cells in response to small sized bovine bone substitute (bio-oss) results in an enhanced early implantation bed vascularization. Ann. Maxillofac. Surg..

[63] Barbeck M., Booms P., Unger R., Hoffmann V., Sader R., Kirkpatrick C.J., Ghanaati S. (2017). Multinucleated giant cells in the implant bed of bone substitutes are foreign body giant cells—New insights into the material—Mediated healing process. J. Biomed. Mater. Res. A.

[64] Barbeck M., Dard M., Kokkinopoulou M., Markl J., Booms P., Sader R.A., Kirkpatrick C.J., Ghanaati S. (2015). Small-sized granules of biphasic bone substitutes support fast implant bed vascularization. Biomatter.

[65] Barbeck M., Serra T., Booms P., Stojanovic S., Najman S., Engel E., Sader R., Kirkpatrick C.J., Navarro M., Ghanaati S. (2017). Analysis of the in vitro degradation and the in vivo tissue response to bi-layered 3d-printed scaffolds combining pla and biphasic pla/bioglass components–guidance of the inflammatory response as basis for osteochondral regeneration. Bioact. Mater..

[66] Brown B.N., Ratner B.D., Goodman S.B., Amar S., Badylak S.F. (2012). Macrophage polarization: An opportunity for improved outcomes in biomaterials and regenerative medicine. Biomaterials.

[67] McNally A.K., Anderson J.M. (2011). Macrophage fusion and multinucleated giant cells of inflammation. Cell Fusion in Health and Disease.

[68] Ghanaati S., Barbeck M., Orth C., Willershausen I., Thimm B.W., Hoffmann C., Rasic A., Sader R.A., Unger R.E., Peters F. (2010). Influence of beta-tricalcium phosphate granule size and morphology on tissue reaction in vivo. Acta Biomater..

[69] Ghanaati S., Barbeck M., Detsch R., Deisinger U., Hilbig U., Rausch V., Sader R., Unger R.E., Ziegler G., Kirkpatrick C.J. (2012). The chemical composition of synthetic bone substitutes influences tissue reactions in vivo: Histological and histomorphometrical analysis of the cellular inflammatory response to hydroxyapatite, beta-tricalcium phosphate and biphasic calcium phosphate ceramics. Biomed. Mater..

[70] Barbeck M, Jung O, Wenisch S, Schnettler R. Pro- and anti-inflammation are needed for synchronous defect healing and degradation of bone substitutes. J. Biomed. Mater. Res. A.

[71] Mittal M., Siddiqui M.R., Tran K., Reddy S.P., Malik A.B. (2014). Reactive oxygen species in inflammation and tissue injury. Antioxid. Redox Signal..

[72] Burchardt H. (1983). The biology of bone graft repair. Clin. Orthop. Relat. Res..

[73] Hing K.A. (2005). Bioceramic bone graft substitutes: Influence of porosity and chemistry. Int. J. Appl. Ceram. Technol..

[74] Lindahl P., Hellstrom M., Kalen M., Betsholtz C. (1998). Endothelial-perivascular cell signaling in vascular development: Lessons from knockout mice. Curr. Opin. Lipidol..

[75] Oh S.J., Kurz H., Christ B., Wilting J. (1998). Platelet-derived growth factor-b induces transformation of fibrocytes into spindle-shaped myofibroblasts in vivo. Histochem. Cell Biol..

[76] Kouri J., Ancheta O. (1972). Transformation of macrophages into fibroblasts. Exp. Cell Res..

[77] Iwano M., Plieth D., Danoff T.M., Xue C., Okada H., Neilson E.G. (2002). Evidence that fibroblasts derive from epithelium during tissue fibrosis. J. Clin. Investig..

[78] Campbell J.H., Efendy J.L., Han C., Girjes A.A., Campbell G.R. (2000). Haemopoietic origin of myofibroblasts formed in the peritoneal cavity in response to a foreign body. J. Vasc. Res..

[79] McAnulty R.J. (2007). Fibroblasts and myofibroblasts: Their source, function and role in disease. Int. J. Biochem. Cell Biol..

[80] Morais J.M., Papadimitrakopoulos F., Burgess D.J. (2010). Biomaterials/tissue interactions: Possible solutions to overcome foreign body response. AAPS J..

[81] Wang H., Li Y., Zuo Y., Li J., Ma S., Cheng L. (2007). Biocompatibility and osteogenesis of biomimetic nano-hydroxyapatite/polyamide composite scaffolds for bone tissue engineering. Biomaterials.

[82] Das D., Zhang Z., Winkler T., Mour M., Günter C.I., Morlock M.M., Machens H.-G., Schilling A.F. (2011). Bioresorption and degradation of biomaterials. Tissue Engineering III: Cell-Surface Interactions for Tissue Culture.

